# Som-Based Class Discovery Exploring the ICA-Reduced Features of Microarray Expression Profiles

**DOI:** 10.1002/cfg.444

**Published:** 2004-12

**Authors:** Andrei Dragomir, Seferina Mavroudi, Anastasios Bezerianos

**Affiliations:** Medical School University of Patras Patras GR-26500 Greece

## Abstract

Gene expression datasets are large and complex, having many variables and unknown internal structure. We apply independent component analysis (ICA) to derive a less redundant representation of the expression data. The decomposition produces components with minimal statistical dependence and reveals biologically relevant information. Consequently, to the transformed data, we apply cluster analysis (an important and popular analysis tool for obtaining an initial understanding of the data, usually employed for class discovery). The proposed self-organizing map (SOM)-based clustering algorithm automatically determines the number of ‘natural’ subgroups of the data, being aided at this task by the available prior knowledge of the functional categories of genes. An entropy criterion allows each gene to be assigned to multiple classes, which is closer to the biological representation. These features, however, are not achieved at the cost of the simplicity of the algorithm, since the map grows on a simple grid structure and the learning algorithm remains equal to Kohonen’s one.


**Comparative and Functional Genomics**
*Comp Funct Genom* 2004; **5**: 596–616.
Published online in Wiley InterScience (www.interscience.wiley.com). **DOI:** 10.1002/cfg.444

**Research Paper**

### SOM-based class discovery exploring the ICA-reduced features of microarray expression profiles

Andrei Dragomir*, Seferina Mavroudi and Anastasios Bezerianos
*Medical School, University of Patras, GR-26500 Patras, Greece*

**Correspondence to:
Andrei Dragomir, Medical School, University of Patras, GR-26500 Patras, Greece.
E-mail:
adragomir@heart.med.upatras.gr*

### Abstract

**Gene expression datasets are large and complex, having many variables and unknown internal structure. We apply independent component analysis (ICA) to derive a less redundant representation of the expression data. The decomposition produces components with minimal statistical dependence and reveals biologically relevant information. Consequently, to the transformed data, we apply cluster analysis (an important and popular analysis tool for obtaining an initial understanding of the data, usually employed for class discovery). The proposed self-organizing map (SOM)-based clustering algorithm automatically determines the number of 'natural' subgroups of the data, being aided at this task by the available prior knowledge of the functional categories of genes. An entropy criterion allows each gene to be assigned to multiple classes, which is closer to the biological representation. These features, however, are not achieved at the cost of the simplicity of the algorithm, since the map grows on a simple grid structure and the learning algorithm remains equal to Kohonen's one. Copyright © 2005 John Wiley & Sons, Ltd.**

**Keywords:** microarrays; clustering; class discovery; self-organizing maps; independent component analysis

**Supplementary material for this article can be found at: http://www.interscience.wiley.com/jpages/1531-6912/suppmat/**

Accepted: 19 November 2004

### Introduction

High-throughput methods developed in the last decade that allow measuring and recording large amounts of data have offered new directions to biological and medical research. Transcriptional profiling using microarray technology makes possible the extraction of whole-genome expression data, which is in need of suitable analysis tools. Starting from virtually no literature a few years ago, this field has come to dominate many conferences and journals (Kohane *et al*., 2003).

Genes involved in different biological processes that are being affected as a result of experimental treatment exhibit specific patterns of variation in their expression. Therefore, genes' expression *profiles*[1] reflect genes responses to a particular set of experiments. Clearly, microarray measurements contain information about many different aspects of gene regulation and function. It is believed, and previous studies confirmed these assumptions (Lieberman, 2001; Lee *et al*., 2003), that by suitably decomposing the gene expression data, resulting components may correspond to distinct biological functions and the decomposition may help producing a less redundant representation of data.

Methods like Principal Component Analysis (PCA) and Independent component analysis (ICA) are usually employed to represent the original data in a manner that facilitates subsequent analysis

[1] In the context of the current paper, by "*profile*" we refer to the expression values of a gene recorded over a set of experiments.

Copyright © 2005 John Wiley & Sons, Ltd.

by means of a suitable transformation[2]. However, as PCA is only a second-order method (i.e. it finds representations of the data using information only in the co-variance matrix) and constrains the direction vectors to be orthogonal, we can just decorrelate the components. ICA imposes statistical independence on the individual output components, using higher-order statistics (e.g. kurtosis), which means information not contained in the co-variance matrix. If the underlying components are Gaussian, it is necessary to use PCA (Gaussian distributions can be completely determined by the information contained in the co-variance matrix), since the ICA model imposes the restriction of non-Gaussianity on all the components (with the possible exception of one). However, for non-Gaussian data, ICA should be employed, since PCA representations may lack important intrinsic information (Karhunen *et al*., 1997). Recent studies suggest that the distribution of gene expression data deviate from the Gaussian distribution and therefore ICA can be effectively applied in order to reveal biological relevant features (Kreil *et al*., 2003; Liebermeister, 2001).

In this study we employ independent component analysis (ICA), due to its capability to take into account higher-order dependencies in the data. The linear ICA is a method for the construction of a linear non-orthogonal coordinate system, in which the components are required to be statistically independent. However, it must be noted at this point that many practical implementations of ICA find components with minimal statistical independence (which is the case for our work also). The consideration of higher-order statistics in ICA allows meaningful information within the data to be exploited. Its capability of estimating underlying sources from an observed dataset has been successfully tested in blind-source separation problems, where the source signals are extracted from an observed data consisting of a mixture of the original signals. Since the expression of genes is controlled by a combination of underlying processes that regulate the way cells behave, it is assumed that ICA can be a useful tool for finding a meaningful representation of the gene expression data. The results of Liebermeister *et al*. (2002) have shown that the retrieved independent components correspond to putative biological processes in yeast cell cycles and may reveal characteristic differences between cell types.

Both PCA and ICA have been previously used in gene expression analysis as either a dimensionality reduction method only (Yeung *et al*., 2001b) or as a method to obtain a more meaningful representation of the data, and subsequently to analyse the biological significance of its decomposition (Liebermeister, 2001). In addition, simple clustering of the decomposed data was performed, in order to improve the prediction of functional classes for gene expression datasets with incomplete annotation (Lee *et al*., 2003).

Clustering of gene expression data, i.e. the grouping of genes into biologically meaningful groups according to their expression profile, is a good starting point for the analysis of a genome whose function remains largely unknown at this time. The simple underlying assumption in all gene-clustering techniques is that genes that appear to be expressed in a similar manner (have similar expression profiles) are in fact involved in the same process and share similar biological functions. The corollary to this assumption is that, although genes may distantly affect the function of other gene products, they fall into groups of more tightly regulated biological processes.

Different clustering techniques have previously been applied directly to microarray data (Brazma *et al*., 2002; Eisen *et al*., 1998; Mavroudi *et al*., 2002; Papadimitriou *et al*., 2001). Although the first clustering methods applied were mostly developed outside biological research, such as hierarchical clustering and K-means, they still revealed biologically relevant information. However, some of their characteristics are not suited for their use in clustering expression data. Perhaps the strongest impact on the final results of some methods is the necessity of specifying the expected number of clusters, as in the case of K-means, as well as the classic self-organizing map (SOM), a number that is almost impossible to predict in advance (Moreau *et al*., 2002). In the case of greedy hierarchical clustering, major drawbacks are the impossibility of relocating objects that may have been incorrectly clustered at an early stage, as well as solution non-uniqueness (the solution may be dependent on the order of presentation of the data to the algorithm) (Morgan *et al*., 1995).

[2] Although the methods discussed can be extended to non-linear variants, we treat only linear transformations in this paper.

Copyright © 2005 John Wiley & Sons, Ltd. 

Recently, many new clustering algorithms have started to tackle some of the limitations of the above-mentioned earlier methods, e.g. the self-organizing tree algorithm (Campos Marcos *et al.*, 2001), but still work needs to be done. Based on our previous work on clustering of gene expression data (Mavroudi *et al.*, 2002), we intended to develop a new method addressing a number of shortcomings of both classical and the newer clustering methods, more specific to microarray data.

We propose to use clustering of the gene expression profiles in the component space resulting from ICA decomposition. After decomposition, the original gene expression data is represented by a new set of variables with respect to a new set of basis vectors. Our method is based on the self-organizing map of Kohonen (2001), since the standard SOM algorithm has a number of properties that render it a candidate of particular interest as a basic framework for building more advanced algorithms for clustering applications. SOMs can be implemented easily, are fast, robust, and scale well to large datasets. They allow one to impose partial structure on the clusters and facilitate visualization and interpretation. In cases where hierarchical information is required, it can be implemented on top of a SOM, as by Vesanto *et al.* (2000). We modified the standard SOM algorithm in order to account for the following peculiarities of gene expression data, which could not be handled by the basic SOM:

1. The number of gene clusters is unknown, although the SOM requires a predefinition of this number.
2. Closely related to using a predefined number of clusters is the consideration that even genes which are not really co-expressed with other cluster members (and therefore represent noise) are forced to end up in one of the clusters, and thereby hamper the further analysis of the clusters.
3. The outcome of the clustering with the SOM relies completely on the distance between genes and the map nodes. However, it is known that genes with related functions do not always show the same high degree of similarity within their respective clusters, i.e. different functional classes have different degrees of heterogeneity. Some clusters may be more compact, while others may be more spread. Prior information about the functional categories of genes could aid the clustering, but is usually ignored.
4. Genes belong to more than one functional category, although the SOM cannot handle multi-labelled data.
5. The number of genes in the different functional categories is unbalanced (e.g. large categories in yeast may contain more than 2200 genes and small categories as few as four genes). This makes it difficult for the basic SOM to map the rare classes into distinct clusters.

It is obvious that the above-mentioned problems are not exclusively SOM-specific, but affect many other clustering algorithms. Thereby their adjustment improves not only over the basic-SOM algorithm (Tamayo *et al.*, 1999) but also over many other existing methods.

There have been several dynamically extendable schemes proposed recently. The dynamic topology representing structures (Si *et al.*, 2000) adaptively grows the number of output nodes by applying a vigilance test; self-organized tree algorithms (Campos *et al.*, 2001; Herrero *et al.*, 2001) are tree-building algorithms that use unsupervised learning to construct hierarchical representations of the data; the joint entropy maximization approach (Van Hulle *et al.*, 2002) employs kernel methods in a learning algorithm that forms topological maps; Azuaje (2001) uses a two-layer neural network structure that implements fuzzy logic operations in order to achieve a number of pattern-matching and adaptation formations. The growing cell structures algorithm (Fritzke, 1995; Cheng *et al.*, 2001) is based on SOM but replaces the basic two-dimensional grid by a network of nodes whose connectivity defines a system of triangles.

Our approach has similarities to the above-described methods, as it is driven by the same principle of expansion based on locally accumulated error. Perhaps the main difference in our approach is that we include prior functional knowledge in order to guide the map expansion and to design the expansion criteria. Of course, we are aware that in molecular biology classes may be defined but are neither complete nor completely trusted. This shouldn't be a reason to completely neglect the knowledge we have acquired so far, but constitutes a reason for avoiding an analysis which would be too tied to the original classes. In contrast to most supervised approaches, which do not give

Copyright © 2005 John Wiley & Sons, Ltd.

any insight into the internal structure of the data but only classify data into *a priori* given classes, we are still aiming at class discovery, while taking into account any *a priori* knowledge.

Comparing with other SOM-based semi-supervised methods in the literature (Sinkkonen *et al.*, 2000; Kohonen, 2001), while these approaches are aimed for the cases where complete and reliable class information exists, we aim to exploit supervised information only when it exists, and thus we confront the problems caused by incomplete and unreliable class labelling of gene expression data. Additionally, we consider the fact that, due to the possible co-expression of genes with different groups of genes in response to the varying demands of the cell, genes do not always belong to a unique class, but may be multi-labelled. Finally, in contrast to the complexity of some of the mentioned schemes, we built simple algorithms that can be implemented easily and the training of the models is very efficient.

There are several aspects in which the current work improves over our previous method (Mavroudi *et al.*, 2002). A substantial enhancement is provided by our choice to make use of the advantages offered by ICA. The linear transform not only produces suitable representations of the gene expression data by filtering out unwanted sources of variation and easing this way further cluster analysis, but also helps to reveal unobserved variables that highlight particular biological factors. Also of great importance is the effective framework provided for reducing data dimensionality, which has a great impact on the computational complexity of the algorithm. Further improvement of our algorithm is related to an efficient use of multifunctional labelling of the data in a more elaborate entropy-based error measure, as well as from the incorporation of a supervised dimension into our map convergence criteria. These provisions are discussed in detail in the following sections, with current results demonstrating the superiority of the present approach and its potential, not only as a clustering but also as a classification device.

### Methods

A simple representation of gene expression data would be that of a matrix $X \in \Re^{nxm}$, whose rows represent profiles of $n$ genes and the columns contain the genes' expression measurements over $m$ conditions. Each gene profile has a class label, describing its gene's biological function. Usually the data dimensions are very large (thousands to tens of thousands $\times$ tens to hundreds), therefore making it a challenge for the data analysis to find a suitable representation of the data such that the transformed variables give information otherwise hidden in the dataset.

Decomposition refers to a process that transforms the input data space into a space designed in such a way as to allow a meaningful representation of the data, which captures most of the intrinsic information content. Usually, therefore, the input data space undergoes a dimensionality reduction as the projected space is constructed (since redundant information is eliminated). More formally, this means that each $m$-dimensional data vector $\mathbf{x}$ of the original data space can be represented by $d$ numbers, with $d < m$.

### Gene expression with ICA

Using ICA decomposition we can model the gene expression data matrix as a matrix product:

$$\mathbf{X} = \mathbf{S}\cdot\mathbf{A} \text{ with } x_{ij} = \sum_{l=1}^{m} s_{il} a_{lj} \text{ where}$$

$$i = 1 \ldots n \text{ and } j = 1 \ldots m \quad (1)$$

where $\mathbf{S}$ is the matrix containing the data representation in independent component space. The model represents the original data by new variables (columns of $\mathbf{S}$) or, alternatively, seen with respect to a new basis formed by the rows of the mixing matrix $\mathbf{A}$. The gene expression profiles $\mathbf{x}_i$ will be represented in component space by the profiles $\mathbf{s}_i$ (rows of $\mathbf{S}$), which will inherit the class labels from the correspondent $\mathbf{x}_i$. The new set of variables, the independent components, have minimal statistical independence between them and may be interpreted as influences of some unobserved variables, as described by matrix $\mathbf{A}$. The task of ICA is to estimate from the observed gene expression data the independent components or, equivalently, to estimate the mixing matrix $\mathbf{A}$.

Furthermore, we are specifically interested in extracting most of the information contained in the original gene expression data within just a few

Copyright © 2005 John Wiley & Sons, Ltd.

independent components. ICA offers an effective framework for dimensionality reduction. The procedure we adopt for truncating the profiles $\mathbf{s}_i$ in order to keep only independent components that are important in our analysis is presented in Results. Intuitively, similar experiments/conditions from the original gene expression set could be described by a single component in the transformed dataset. After retaining the most informative components, we will use as inputs to our algorithm the truncated profiles $\mathbf{s}_i$, which will be referred to from now on as *patterns*.

### Approaches to ICA

One main category of approaches to the ICA computation relies upon batch computations that minimize or maximize some relevant contrast functions. These algorithms suffer from the problem that they require very complex matrix operations. Another category considers adaptive algorithms that are based on stochastic gradient algorithms, implemented with neural networks. These approaches suffer from slow convergence. Moreover, the convergence itself depends critically on the choice of the learning rate parameters and therefore it cannot be guaranteed.

On the other hand, the fixed-point ICA algorithm, presented by Hyvarinen *et al.* (1997), is an approach that utilizes a highly efficient fixed-point iteration scheme for finding the local extrema of a suitable contrast function that accepts as arguments a linear combination of the observed variables. The detection of the local extrema of this contrast function makes possible the approximation of the non-Gaussian independent components. The fixed-point algorithm works practically for any non-Gaussian distribution of the independent components (Hyvarinen, 1999) and for any choice of the contrast function. The choice affects only the performance optimization. This is very important, since there is little *a priori* information about the distribution of the gene expression profiles. Additionally, the algorithm in its hierarchical version finds the independent components one at a time, instead of working in parallel like most of the suggested ICA algorithms that solve the entire mixing matrix. This makes it possible to estimate only certain desired independent components (Hyvarinen *et al.*, 1997).

Before applying the fixed-point algorithm, a preliminary *whitening* of the data $\mathbf{x}$ is performed, a useful preprocessing step in data analysis. The observed vector $\mathbf{x}$ is linearly transformed to a vector $\mathbf{u} = \mathbf{M} \bullet \mathbf{x}$, such that the elements $u_i$ are mutually uncorrelated and all have unit variance. Therefore, the correlation matrix of $\mathbf{u}$ equals unity: $E\{\mathbf{u}^{\mathrm{T}} \bullet \mathbf{u}\} = \mathrm{I}$. This transformation can be accomplished effectively with PCA. A benefit of using PCA is that in addition to whitening, it can at the same time perform an effective dimensionality reduction of the data. Specifically, the dimension of the transformed data vector $\mathbf{u}$ is reduced to $d$, where $d$ is the number of independent components that we wish to obtain. Therefore, after the transformation we have:

$$\mathbf{u} = \mathbf{M} \bullet \mathbf{x} = \mathbf{M} \bullet \mathbf{A} \bullet \mathbf{s} \quad (2)$$

Since the components $s_i$ are independent by assumption:

$$E\{\mathbf{u}^{\mathrm{T}} \bullet \mathbf{u}\} = \mathbf{B}E\{\mathbf{s} \bullet \mathbf{s}^{\mathrm{T}}\}\mathbf{B}^{\mathrm{T}} = \mathbf{B} \bullet \mathbf{B}^{\mathrm{T}} = \mathbf{I} \quad (3)$$

It follows that $\mathbf{B} = \mathbf{M} \bullet \mathbf{A}$ is an orthogonal matrix. Therefore, the problem of finding an arbitrary full-rank matrix $\mathbf{A}$ is reduced after the PCA whitening to the simpler problem of finding an orthogonal matrix $\mathbf{B}$, from which the independent components are obtained with $\mathbf{s} = \mathbf{B}^{\mathrm{T}} \bullet \mathbf{u}$. The criterion for finding the orthogonal matrix $\mathbf{B}$ is to minimize the final objective function $J(\mathbf{w}) = E\{(\mathbf{w}^{\mathrm{T}}\mathbf{u})^4\} - 3\|\mathbf{w}\|^4$, where $\mathbf{w}^{\mathrm{T}}\ \mathbf{u}$ are linear combinations of the observations $u_i$ and with $\mathbf{z} = \mathbf{B}^{\mathrm{T}}\ \mathbf{w},$ with $\|\mathbf{w}\| = \|\mathbf{z}\| = 1$, as in Hyvarinen *et al.* (1997).

### The SOM-based learning algorithm

### Algorithm dynamic growing and adaptation

Unlike the standard SOM, which has a fixed architecture, we designed a model initialized with a map that consists of only four nodes, arranged on a rectangular $2 \times 2$ grid, and then expands automatically in order to properly represent the input data. Each node corresponds to a different cluster, with one or several clusters in the final map configuration representing a class. Weights of the starting nodes are initialized with random patterns from the input dataset. Patterns representing the gene profiles in the independent component space are successively presented to the map, each of them being assigned

Copyright © 2005 John Wiley & Sons, Ltd. 

to the node with closest weight vector, according to a distance measure (named 'winner node') that is consequently adjusted together with its direct neighbours, to represent the patterns mapped. Since the map starts with a much smaller than usual sized SOM, there is no requirement for a large neighbourhood to train the whole map at the first learning steps (e.g. with four nodes initially in the map, a neighbourhood of one only is required). As training proceeds, during subsequent training runs, the area defined by the neighbourhood becomes localized near the winning node, not by shrinking the vicinity radius (as in the standard SOM) but by enlarging the SOM with the dynamic growing.

The learning rule for the adjustment of the weight vector remains the same as in the standard SOM. Specifically, during the adaptation phase the weight vectors of the four nodes in the direct neighbourhood of the winner and for the winner itself according to the following formula:

$$\mathbf{w}_j(t+1) = \begin{Bmatrix} \mathbf{w}_j(t), & j \notin N_t \\ \mathbf{w}_j(t) + \mu_{\varpi}\cdot\Lambda(d(i,j))\cdot\Delta\mathbf{w}_j(t), & j \in N_t \end{Bmatrix}$$

where the learning rate $\mu_\omega(t)$ is an exponentially decreasing sequence of positive parameters, $N_t$ is the neighbourhood of the winner node at the $t$th learning step, $\Lambda(d(i,j))$ is the neighbourhood function implementing different adaptation rates even within the same neighbourhood, $\Delta\mathbf{w}_j(t)$ is the distance between the input pattern and its closest weight vector and $d(i,j) = ((i_r - j_r)^2 + (i_c - j_c)^2)^{\frac{1}{2}}$ (the row and column of a node $i$ are denoted by $i_r, i_c$, respectively).

The learning rate $\mu_\omega(t)$ typically starts with a value of 0.1 and decreases exponentially to 0.02. These values are chosen as to have a relatively fast convergence without, however, sacrificing the stability of the map (Haykin, 1999). The learning rate should be kept to a small value during the convergence phase, but not allowed to decrease to zero, in order to avoid the network getting stuck to a map configuration with topological defects.

The neighbourhood function $\Lambda(d(i,j))$ can be defined with the following simple formula:

$$\Lambda(d(i,j)) = \begin{cases} 1 & \textit{if}\ i = j \\ \alpha \quad 0 < \alpha < 1\ \textit{if} & (i_r - j_r)^2 + (i_c - j_c)^2 = 1 \\ 0 & \textit{otherwise} \end{cases}$$

The expansion of the map is driven by a process that aims to minimize two concepts of error, the *quantization error* (representing the unsupervised part of the learning algorithm) and the *classification error* (representing the supervised part). The relative significance of the supervised part is controlled by a parameter $r_{su}$. More formally, we could state that the learning and the expansion of the map aim to minimize a measure $\tau_E$ of the form:

$$\tau_E = \sum_{i=1}^{N_d} \varepsilon_i \tag{4}$$

where $N_d$ is the number of nodes and $\varepsilon_i$ the individual composite error term for node $i$, defined below:

$$\varepsilon_i = (1 - r_{su})\cdot \textit{UnsupervisedComponent}_i + r_{su}\cdot \textit{SupervisedComponent}_i \tag{5}$$

The map expansion procedure is performed by inserting new nodes in the neighbourhood of the node $j$, with the largest composite error measure after the presentation of all the patterns to the map. Training efficiency and implementation simplicity were motivations for expanding mostly from the boundary nodes (see Figure 1). By these means, depending on the position of the node $j$, the insertion process follows the guidelines described bellow:

1. If node $j$ is a boundary node, the expansion is performed by acquiring one to three new nodes in the direct neighbourhood of the node $j$. The weights of the new nodes are initialized so as to preserve the trend of the weights of neighbouring nodes (to respect the *weight flow*). Specifically, the weight of the new node is computed as $W_N \equiv W_{r,c-1} = W_{r,c} + (W_{r,c} - W_{av,c})$, with $w_{av,c}$ denoting an average of the weights of nodes in the neighbourhood of the node initiating the expansion.
2. If node $j$ is a near boundary node i.e. a node from which the boundary of the map can be reached by traversing in any direction at most two nodes, a percentage (usually 20–50%) of the weight of the node initiating the expansion is shifted towards the outer nodes. This operation alters locally the Voronoi regions and usually, with a few 'rippling' operations, the respective

Copyright © 2005 John Wiley & Sons, Ltd.

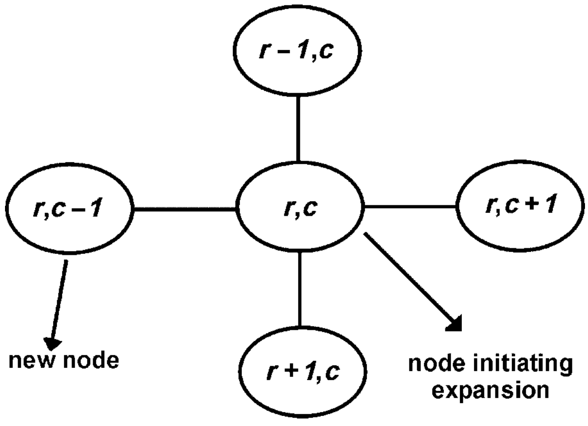

**Figure 1.** Map expansion by boundary node insertion. New nodes are added in the free positions in the grid around the node initiating the expansion (so-called *winner node*). The weight of the new node ($w_{r,c-1}$) is initiated with a value representing a local average of the neighbouring nodes, maintaining the *weight-flow* (i.e. if $\mathbf{w}_{r,c} > \mathbf{w}_{r,c+1}$, then $\mathbf{w}_{r,c-1} > \mathbf{w}_{r,c}$)

node's weight is propagated to a boundary node (located nearby).

3. Finally, if the node with the largest error measure is neither placed on the boundary nor near the boundary, the alternative of inserting a whole column of new nodes is used. The rippling of the weights is avoided in these cases, since usually excessive computation times are required before the weights propagate from a node placed deep in the map to a boundary node. The column of new nodes is inserted in the grid respecting the direction of largest total error. Specifically, the sum of the composite errors for the nodes in the grid columns to the right and to the left of the winner node is computed. The new column of nodes is inserted to the side where the error measure is larger. The initial weight values of the new nodes are assigned with respect to the weight flow of the neighbouring node weights, as described above.

Map expansion, followed by the re-adaptation of the weight vectors, is performed repeatedly until convergence (based on the relative change of the total error measure for all nodes between successive training runs) and expansion criteria are met. These criteria are discussed in the following section. After the final expansion of the map is performed, we proceed to a fine-tuning of the respective map configuration. The procedure is similar to the adaptation phase, with the exception that the learning rate decreases to a smaller value in order to allow fine adjustments to the final structure and to allow a lower convergence threshold to be imposed.

### Expansion mechanisms

As introduced above, the map expansion takes into account two kinds of error measures. Minimizing the unsupervised part of the above composite error measure $\varepsilon$ corresponds to producing clusters of genes that are similar to each other according to a similarity measure (we prefer to use the Manhattan distance, since it is less sensitive to outliers). This term assures the proper functioning of the algorithm, even for completely unlabelled data. The minimization of the supervised component of the error measure, on the other hand, secures a proper use of the labelling. Minimal supervised error forces patterns with similar labels into the same cluster.

### Unsupervised component of the composite error measure

We have modified the local error measure that is commonly used for implementing dynamically growing schemes (Alahakoon *et al.*, 2000; Herrero *et al.*, 2001). The measure we are using is customized in such a way to be less sensitive when a large number of similar patterns are mapped to the same node. The unsupervised term in (5) is, thus, a weighted average local error:

$$\varepsilon_{wale}(i) = \frac{1}{|S_i|} \sum_{x \in S_i} rc_x \cdot Dist(\mathbf{x}, \mathbf{w}_i) \qquad (6)$$

where $S_i$ denotes the set of patterns $\mathbf{x}$ mapped to node $i$, $|S_i|$represents the number of elements of the set $S_i$, $\mathbf{w}_i$ is the weight vector of the node $i$ and the Dist operator, the distance metric. The weight factor $rc_x$ is given by:

$$rc_x = \frac{1}{L_x} \sum_{l=1}^{L_x} \left( \frac{\#\ patterns\ of\ class\ c_l}{\#\ total\ patterns} \right)^{-1/2} \qquad (7)$$

and corresponds to the average frequency ratio of the $L_x$ classes $c_l$ to which pattern $\mathbf{x}$ belongs. If a pattern $\mathbf{x}$ is unlabelled (i.e. is not assigned to any functional class) $rc_x$ is set to $rc_x = 1$. The

Copyright © 2005 John Wiley & Sons, Ltd. 

reason for choosing the exponent factor $-1/2$ is to make the errors on the low frequency classes account more (e.g. if class A is 100 times less frequent than B, it is 'amplified' 10 times more). By this means it promotes an additional expansion in the neighbourhood of the node where rare classes are mapped, and consequently it enhances the possibility that rare classes will be mapped on distinct nodes. As a result, the representation of these low-frequency classes is improved. We should note that these classes are usually of most biological significance. Being an average value, $\varepsilon_{wale}$ does not increase by the accumulation of a large number of similar patterns to a node.

### Supervised component of the composite error measure

Each node $i$ is assigned a *classification vector* **cl** with elements $cl_k, k = 1 \ldots N_c$, where $cl_k$ is the ratio of the patterns with functional label $k$ among all the patterns mapped to the node $cl_k = \frac{V_k}{V_{pattern}}$ ($N_c$ is the total number of classes, and unlabelled patterns do not contribute to $V_{pattern}$). The vector **cl** is considered as the predicted soft classification and each $cl_k$ quantifies the degree of certainty that the node $i$ (and consequently the mapped patterns) is assigned a label $k$.

We defined an *entropy-like* parameter, which quantifies the uncertainty of the class label of node $i$, similarly to an entropy measure (Haykin, 1999). However, since each pattern may have multiple functional labels, for a node $i$, we may have the situation where the sum $\sum_{k=1}^{N_c} cl_k > 1$. As a result, we cannot interpret $cl_k$ as a probability (that the node $i$ is assigned a label $k$) distribution and therefore the entropy parameter we defined using this ratios is not a proper entropy. Additionally, we compute for each $cl_k$ value a corresponding $q_k$ value, such as $q_k = 1 - cl_k$, which may be interpreted as a measure indicating the degree in which node $i$ does not belong to class $k$, so that obviously $cl_k + q_k = 1$. The additional parameter $q_k$ helps in giving a better account of the class uncertainty within nodes. Then, the entropy-like parameter can be computed as the sum of the 'entropies' of all individual classes:

$$Hl(i) = -\sum_{k=1}^{N_c} cl_k \cdot \log cl_k + q_k \cdot \log q_k \qquad (8)$$

This entropy-like quantity retains properties similar to the entropy. *Hl(i)* is zero for unambiguous nodes (nodes that contain patterns with the same functional label) and increases as the uncertainty about the class label of the respective node increases. The upper bound of *Hl(i)* is $N_c \log(2)$ and corresponds to the situation where all the classes are equiprobable (i.e. the labelling mechanism does not favour a particular functional class).

The effective handling of the multi-labelled data can be explained by a simple example. Let $N_c = 2$ and suppose that 50 patterns are assigned to node $i$, all of them having as a label *both* classes, while another 100 patterns are assigned to node $j$, half of them belonging to one class and the other half to the other. Although in each case there are 50 patterns voting for each class, the quantity *Hl(i)* will be high for node $j$ ($Hl(j) = 2\log(2)$) and zero for node $i$ ($Hl(i) = 0$). Thus, it would correctly indicate that node $i$ should be kept as it is, representing patterns which are labelled simultaneously by both classes, and that node $j$ does not unambiguously represent any of the two classes and the map should be expanded in its neighbourhood.

Existing class information is explored in order to resolve better near class boundaries. In other words, since nodes with high entropy correspond to nodes where patterns of different classes are equally mapped, it is intuitively clear that these nodes correspond to regions that lie near class boundaries. This means that by achieving a small entropy term (by adding new nodes near to these regions) proper class boundaries are created.

### Balance between the supervised and unsupervised part

As stated before, the $r_{su}$ parameter controls the relative significance of the supervised information in the analysis of the respective dataset. The parameter could be set manually in advance; however, this affects the performance of the algorithm crucially. With $r_{su} = 0$ we have pure unsupervised learning. The higher the $r_{su}$ value, the more the composite error $\varepsilon$ is minimized for configurations that fit better the *a priori* classification. If the goal of the clustering analysis is to derive class labels for gene patterns with unknown functionality based on already annotated data, the higher the confidence in the annotation of the expression data is, the higher

Copyright © 2005 John Wiley & Sons, Ltd.

the $r_{su}$ parameter should be set. For sufficiently large values of $r_{su}$ the *a priori* component dominates completely. Clearly, since in this case the information within the dataset is suppressed, care should be taken when dealing with such situations, especially if the goal of the analysis is exploratory insight of the dataset.

Therefore we prefer to produce multiple models and to introduce a model selection step, in order to select a well-performing parameter automatically. Specifically, we start with a zero-valued supervision parameter to produce the first purely unsupervised model, and then the whole algorithm is repeated each time for an increased $r_{su}$ parameter until the classification performance is near optimal. The classification performance for each $r_{su}$ value is measured with a specialized soft classification performance measure, *ClassifPerf*($r_{su}$), which is able to handle the multiple labelling of patterns (Sable *et al.*, 1999) and is evaluated as follows: as already mentioned, each node $i$ is assigned a *classification vector* **cl** with elements $cl_k$. Each pattern has also an *a priori* (binary) classification vector, $\mathbf{cl^{pattern}}$. Then, for each class label $k$ of each pattern $j$ mapped to node $i$, a score is assigned. This score equals $cl_k$ if the corresponding label is included in the original class assignment of the pattern $j$ (i.e.$cl_k^{pattern} = 1$) and equals $q_k = 1 - cl_k$ in the other case (i.e. $cl_k^{pattern} = 0$). Intuitively, a small $cl_k \approx 0$ for a class that does not appear as a functional label (i.e.$cl_k^{pattern} = 0$) of an input pattern $j$, is much more a success than a failure, therefore it is considered by a score $q_k \approx 0$. In this way a total score for each pattern $j$ is computed as:

$$TotalScore_j = \sum_{k=1}^{N_C} sc_k$$

$$\text{where } sc_k = \begin{cases} cl_k \; if & cl_k^{pattern} = 1 \\ q_k = 1 - cl_k \quad if & cl_k^{pattern} = 0 \end{cases}$$

The performance $Perf_j$ for each pattern $j$ is then obtained by dividing the $TotalScore_j$ with the total number of functional class labels $N_c$:

$$Perf_j = \frac{TotalScore_j}{N_c}$$

The global measure of the performance $Class-ifPerf(r_{su})$ for a given ratio $r_{su}$ is obtained by averaging the $Perf_j$ values for all the patterns of the dataset *DS*, i.e:

$$ClassifPerf(r_{su}) = \frac{\sum_{i \in DS} Perf_i}{|DS|}$$

with |DS| denoting the number of elements in the dataset.

Finally, a well performing ratio $r_{su}$ is selected by using the following criteria: on the curve of classification performance over $r_{su}$ we select the value of $r_{su}$ for which a significant increase of the classification performance is obtained, followed by a relatively levelled part in the plot. The increase of the classification performance with an increasing value of $r_{su}$ is explained by the increased role of the *a priori* information for the formation of the proper cluster structure. The significance of the levelled part in the region of the plot of classification performance that follows the chosen $r_{su}$ is that further increasing the strength of *a priori* information does not provide an improved generalization performance. The second criterion that guides the identification of the proper value of $r_{su}$ is that, for the chosen value of the supervised parameter, the number of nodes in the map is considerably smaller compared to map configurations with comparable classification performance (thus yielding a model with small complexity).

In a condensed form, the main steps of our algorithm are:

1. Initialize $2 \times 2$ map and set $r_{su} = 0$ (pure supervised learning).
   **Repeat//**develop a series of models each of them corresponding to an increasing value of $r_{su}$.
   **Repeat:**
2. Adaptation phase
3. Expansion phase
   **until** criteria for map expansion are satisfied.
4. Fine tuning adaptation phase.
5. Save map configuration for the current supervised/unsupervised balance $r_{su}$.
6. Compute classification performance for current $r_{su}$.
7. Increase significance of the supervised part (increase $r_{su}$)
   **until** classification performance $\approx$ 1.
8. Model selection step

Copyright © 2005 John Wiley & Sons, Ltd.

#### Map convergence and expansion control criteria

The composite error measure also guides the convergence of the map, with the map assumed to have converged when the relative change of the total error measure for all nodes between successive training runs drops below a threshold value. The setting of the threshold value in the range 0.01–0.02 was indicated by several tests performed on different gene expression datasets. It provides sufficient convergence without excessive computation.

Intuitively, the map expansion should stop when the patterns mapped to the nodes of the map are similar and 'not random' (i.e. unrelated). The problem thus reduces to the definition of similarity between patterns. In our setting, similarity has two aspects, supervised and unsupervised. In the unsupervised case, similarity between patterns is related to the distance between them. In order to treat the problem quantitatively, we set a confidence level $\alpha$ from which we derive a threshold $d_{thr}$ for the distance between patterns, below which two patterns are considered similar. The confidence level $\alpha$ has the meaning that the probability that two patterns allocated to the same node are 'random' (not similar) is lower than $\alpha$, if the distance between them is smaller than the threshold. Obviously, the definition of a statistical confidence level would be only possible if the distribution of the distances between random patterns was known. Practically, although the distribution is unknown, it is easy to approximate it by randomly shuffling all the patterns' elements. This randomization destroys the correlations between different patterns, while it retains the other characteristics of the whole set (e.g. ranges and histogram distribution of values). In this way we compute an approximation of the distribution of the distance between random patterns. Figure 2 illustrates the distributions of the distances between the randomized patterns and the actual patterns from the component space. In this case, by choosing a statistically common confidence level $\alpha = 0.05$, we can determine a lower and a upper threshold for the distance between patterns ($dL_{thr}$ and $dU_{thr}$) and consider the distances in the interval $[dL_{thr} dU_{thr}]$ as random. Taking into account that smaller (larger) distance corresponds to a larger (smaller) correlation, we can state that for distances smaller than $dL_{thr}$ a positive correlation between patterns is implied, while a negative correlation is indicated by distances larger than $dU_{thr}$.

Having determined the distance thresholds, we compute for each node $i$ the ratio of intra-node pairwise distances between patterns which fall beyond the thresholds, i.e.:

$$rd_i = \frac{\#distances\ beyond\ thresholds}{\#distances\ within\ node\ i}$$

We then compute the sum:

$$RD = \frac{1}{K}\sum_{i=1}^{K} rd_i$$

where $K$ denotes the number of clusters. Obviously, for the extreme case that every node contains only patterns with inter-pattern distances fall outside these thresholds, $RD$ takes the value $RD = 0$ (representing the least random state possible), whereas for the opposite extreme case, when the nodes only contain patterns with inter-pattern distances that fall within the thresholds, we have $RD = 1$ (representing the most random state possible).

A purely unsupervised expansion control criterion has been used before in the literature (Herrero *et al.*, 2001; Mavroudi *et al.*, 2002). However, such an approach is reliable only if the distribution of the random patterns was known and not estimated. Also, the definition of randomness in such a case depends only on the similarity of the patterns, i.e. the derived thresholds. Therefore, the similarity thresholds would be the same for all nodes, although there is no reason to believe that the degree of similarity between patterns that belong to the same class is bound to be the same for different classes.

We add to the unsupervised control criterion described above a supervised component, which quantifies the difference between the representation of functional classes in the entire dataset and their representations within different nodes. We would expect that a *random* distribution would allocate the patterns in such a way that the representation of classes in a given node would be similar to the representation of the classes in the initial dataset. If the different classes represented in the dataset were of equal size (contained the same number of patterns), we would expect a random assignment to result in a uniform representation of all classes and the entropy-like measure would be an appropriate measure of randomness (i.e. a high entropy value

Copyright © 2005 John Wiley & Sons, Ltd. 

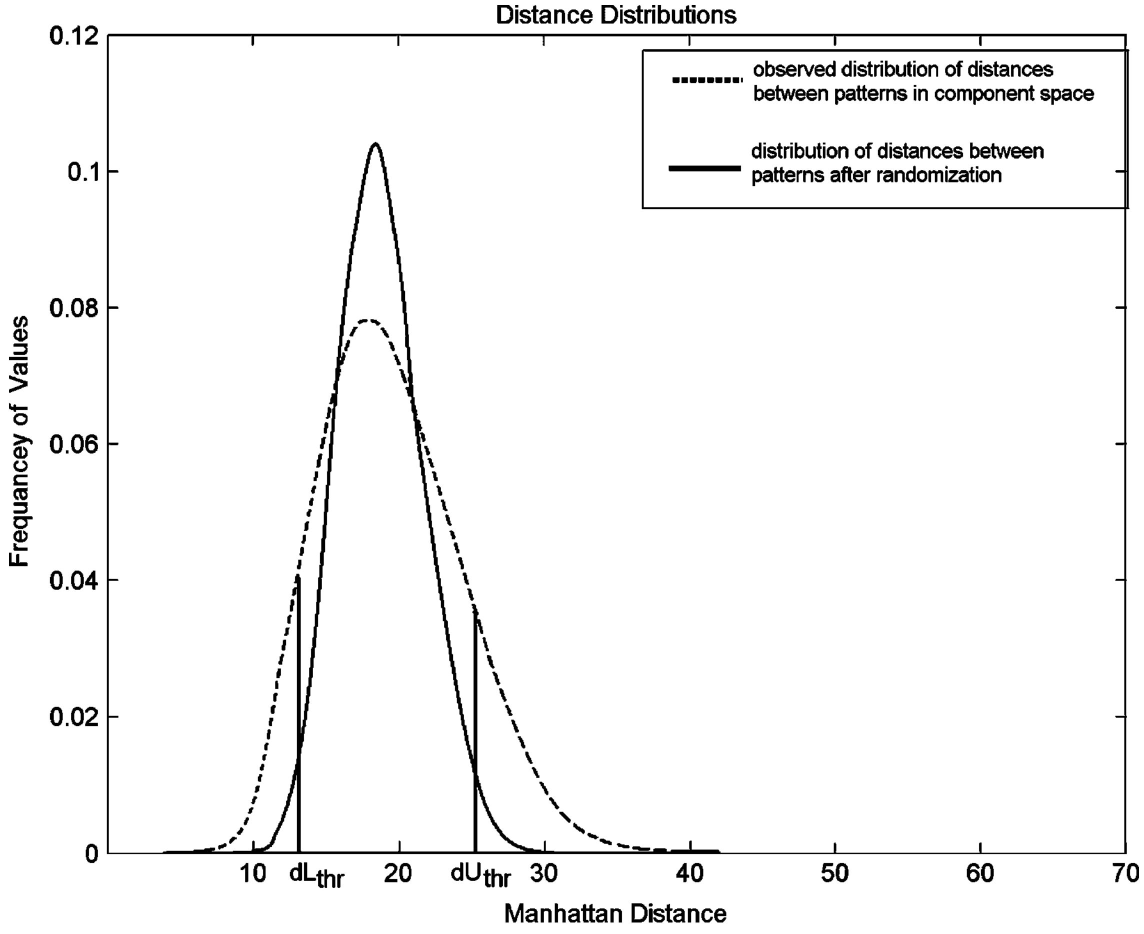

**Figure 2.** The results of the data shuffling illustrate that the distances between randomized data occupy a distinct distribution. The plot concerns the data distributions from dataset 1. $dL_{thr}$ and $dU_{thr}$ are the derived distance thresholds, with similar patterns expected to have inter-pattern distances smaller than $dL_{thr}$

would mean that the distribution is random and a low value that the distribution is not random). However, since most gene expression datasets have a highly unbalanced number of functional classes (with hundreds of genes belonging to a specific functional class, while other classes may be represented by fewer than 10 genes) we prefer to quantify the differences in classes representation in the initial dataset and within each node in terms of a measure based on the Hellinger distance.

The Hellinger distance measures the discrepancy between two *probability density functions* (pdf) and, unlike other divergences (e.g. Kullback–Leibler divergence), it is symmetric, bounded, and satisfies the triangle inequality (we could say that the Hellinger distance is a measure of affinity between two distributions). In a state space $\Omega$, with $\omega \in \Omega$ being the set of all possible states:

$$DH = \sqrt{\sum\nolimits_{\varpi \in \Omega} (\sqrt{p_{\varpi}} - \sqrt{q_{\varpi}})^2} \in [0, \sqrt{2}] \quad (9)$$

Similarly, with the classification vectors defined previously, we define for each node $i$ vectors $\varphi_i$ of the ratios $\varphi_{ik} = \frac{N_{ik}}{N_i}$, where $N_{ik}$ is the number of patterns with functional class label $k$ ($k = 1 \ldots N_c$) mapped to node $i$ and $N_i$ is the total number of labels within node $i$ (i.e. a multi-labelled pattern is counted multiple times, once for each of its labels). Thus, $\varphi_{ik}$ are the observed frequencies of occurrence of patterns with label $k$. One can interpret the $\varphi_{ik}$ as probabilities, since $\sum_{k=1}^{N_C} \varphi_{ik} = 1$. Specifically, for each class $k$, a pattern that is mapped on the node $i$ has a probability of $\varphi_{ik}$ of belonging to

Copyright © 2005 John Wiley & Sons, Ltd. 

the class $k$ and a probability of $\psi_{ik} = 1 - \varphi_{ik}$ of not belonging to the class $k$, with $\varphi_{ik} + \psi_{ik} = 1$. Equivalently, the frequency of occurrence of patterns with class label $k$ in the initial dataset is computed as:

$$\varphi_k^{Data} = \frac{N_P^k}{N_P}$$

with $N_P^k$ being the number of patterns with label $k$ in the initial dataset and $N_P$ being the total number of labels in the dataset (again, multi-labelled patterns count multiple times). Again, $\varphi_k^{Data}$ can be interpreted as probabilities, i.e. the probability that a pattern in the initial dataset belongs to the class $k$. Then, the distance for each node $i$ is given by:

$$DH_i^{Node} = \frac{1}{\sqrt{2} \cdot N_C} \sum_{k=1}^{N_C} \sqrt{\left(\sqrt{\varphi_k^{Data}} - \sqrt{\varphi_{ik}}\right)^2 + \left(\sqrt{\psi_k^{Data}} - \sqrt{\psi_{ik}}\right)^2} \tag{10}$$

where $N_C$ is again the number of distinct classes. The multiplicative factor $\frac{1}{\sqrt{2} \cdot N_C}$ is introduced in order to normalize the distance to the range [0,1]. Finally, we compute the total average distance $TD_H$ for all $K$ clusters as:

$$TD_H = \frac{1}{K} \sum_{i=1}^{K} DH_i^{Node} \tag{11}$$

Combining the unsupervised measure $RD$ previously defined and the supervised distance measure $TD_H$ we derive a total measure of the form:

$$Rand = \beta(1 - TD_H) + (1 - \beta) \cdot RD \quad \in [0, 1] \tag{12}$$

where $\beta$ is a parameter controlling the balance between the supervised and unsupervised term. $Rand = 0$ corresponds to the case where all inter-pattern distances of the clusters fall beyond the thresholds and the distribution of the class labels of the nodes differ completely from their distribution in the initial dataset. Obviously, $Rand = 1$ corresponds to the other extreme case, where the inter-pattern distances and the distribution of the class labels are random.

Finally, the expansion criterion is given by:

$$\textit{Expand map until} : \textit{Rand} \leq \varepsilon \tag{13}$$

The parameter $\varepsilon$ has been determined empirically to 5%: for $\beta = 0$ it has the meaning that we consider the map configuration as *not random* and stops the expansion only if, on average, less than 5% of the patterns which are mapped to the nodes do not fulfil the intra-node threshold requirements. For $\beta = 1$ the configuration is considered as *not random* if, on average, the similarity between the class label distribution of the nodes and the class label distribution of the dataset is less than 5%.

An important observation is that, since the patterns mapped to each cluster fall between certain similarity distance thresholds and have a distinct class label distribution, it follows that patterns that are not tightly co-expressed with other patterns will be mapped to distinct clusters. In other words, nodes that are selected as winners for very few (usually one or two) patterns, termed *uncolonized* nodes, seem to correspond to genes that lack co-expression with other genes and are probably noisy patterns. Of course, there is also a chance that these patterns are not pure artifacts, but very unique patterns that have potential to provide knowledge. Therefore they are amenable to further study, and this is why we do not delete these nodes from our scheme. However, due to the changes in the weight values of the nodes during the map adaptation, some patterns may be accidentally assigned during the training process to different nodes than those to which they are finally assigned. That is why we mark and isolate only the patterns that are consistently (three times or more during the complete map training schedule — for all $r_{su}$ parameter values — consisting of few hundred training runs) mapped to uncolonized nodes. In contrast, nodes that are not selected as winners for any pattern are removed from the map, in order to keep it compact.

### Results

We have performed several experiments to test the performance of our approach on benchmark microarray datasets of the budding yeast *Saccharomyces cerevisiae*, publicly available at the

Copyright © 2005 John Wiley & Sons, Ltd.

Stanford web site. The two (related) datasets were generated by studying this fully sequenced organism with microarrays containing essentially every open reading frame (ORF). The larger dataset contains 80-element gene expression profiles for 6221 genes and the smaller one consists of 79-element gene expression profiles for 2474 genes. Apart from making use of our method's capability of incorporating supervised information into unsupervised learning and assessing its performance in exploratory data analysis, we studied its (re)classification capability by artificially disturbing the functional classification labels in order to assess the strength of the model as a reclassification device. In another experiment, we investigate the meaningful information arising from ICA decomposition. Additional results proving our method's solid performance in clustering, compared to other classical approaches, are presented in the Supplementary Material (**http://www.interscience.wiley.com/jpages/1531-6912/suppmat/**).

### Dataset 1

Samples in this set were collected at various time points during the mitotic cell division cycle (60 time points in four different experiments: $\alpha$-factor, cdc-15, cdc28-based synchronization and elutriation), sporulation experiments (13 time points) and the diauxic shift (seven time points). The sources of these profiles were eight different microarray experiments (Eisen *et al.*, 1998).

The study of yeast genes during the diauxic shift, for example, were obtained from DeRisi *et al.* (1997). With a fluorescence ratio method, they measured the relative abundance of mRNA for the entire yeast genome, to examine the changes in expression that take place with the metabolic shift from anaerobic to aerobic metabolism. The levels of expression of genes were measured in seven samples, taken at 2 h intervals, and reflect metabolic reprogramming that occurred during the diauxic shift.

Annotation for the genes in this dataset was derived from the Functional Classification Catalogue of the Munich Information Center for Protein Sequences (MIPS) Comprehensive Yeast Genome Database (CYGD), available at **http://mips.gsf.de/proj/yeast/CYGD/db/index.html**. The selected annotation included all the 19 *top-level functional categories*. Table 1 presents the functional categories, the number of gene expression profiles (or, alternatively, the number of component profiles $s_i$) corresponding to each annotation and the distribution of the class labels in the gene expression dataset.

**Table 1.** Functional categories in dataset 1

| Functional category | $N_P^i$ | $p_i^{Data}$ |
|---|---|---|
| Metabolism* | 1059 | 0.136 |
| Cell fate* | 423 | 0.054 |
| Cell rescue and virulence | 273 | 0.035 |
| Cellular communication signal transduction mechanism* | 59 | 0.007 |
| Cellular transport and transport mechanism | 480 | 0.062 |
| Energy | 241 | 0.03 |
| Cell cycle and DNA processing* | 620 | 0.079 |
| Protein fate-folding modification destination | 588 | 0.076 |
| Protein synthesis | 346 | 0.044 |
| Regulation of interaction with cellular environment | 194 | 0.025 |
| Control of cellular organization* | 205 | 0.026 |
| Protein activity regulation | 13 | 0.02 |
| Protein with binding function or co-factor requirement | 4 | 0.0005 |
| Subcellular localization | 2206 | 0.284 |
| Transport facilitation* | 305 | 0.039 |
| Transcription | 754 | 0.097 |
| Transposable elements—viral and plasmid proteins | 10 | 0.001 |
| Classification not yet clear | 115 | — |
| Unclassified proteins | 2186 | — |

The first column contains the category name, the second column the number of patterns corresponding to each annotation and the third column, the distribution of the class labels in the dataset.
* Marked categories are employed in the classification validity experiments.

The gene expression profiles are arranged in a table with rows corresponding to genes and columns to the individual log-transformed gene expression ratios of each gene in a particular experimental condition represented by the column. The weighted K-nearest neighbours imputation method presented in Troyanskaya *et al.* (2001) is applied in order to systematically fill up the missing values.

The implementation of the ICA model has been accomplished with a fixed-point algorithm, using an implementation that is freely available at **http://cis.hut.fi/projects/ica/fastica/fp.shtml**. Dimension control by PCA was used and 65 dominant components out of 80 were selected, which accounted for 98.64% of the variance in the

Copyright © 2005 John Wiley & Sons, Ltd.

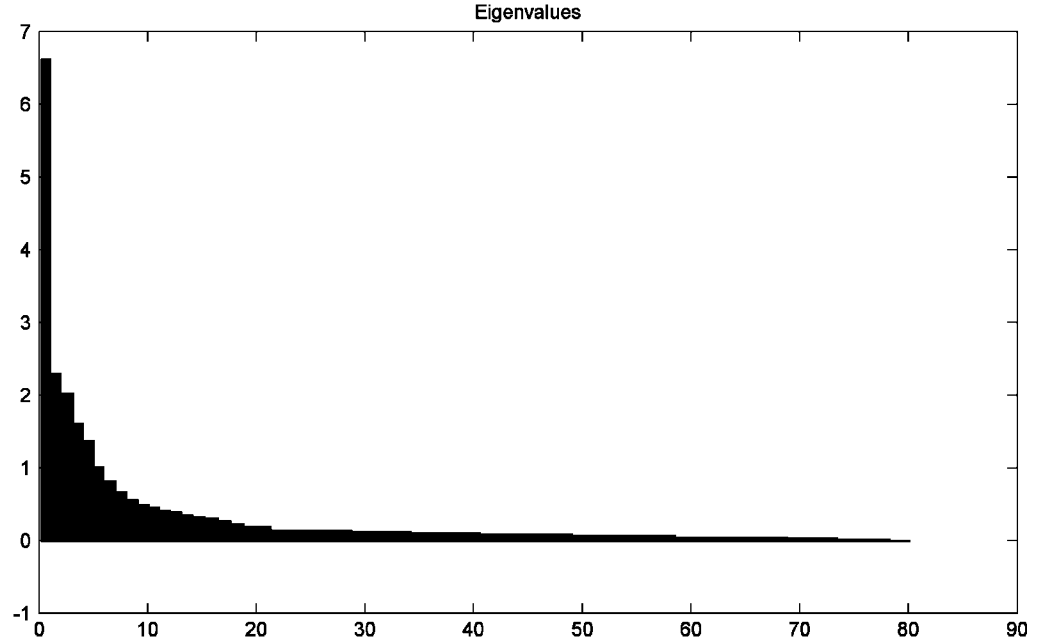

**Figure 3.** Plot of the ordered PCA components obtained from dataset 1. It can be noticed that the first few components contain most of the variance (first 65 components contain 98.64% of the variance in the data)

data. In Figure 3 the eigenvalues corresponding to these principal components are plotted along their indices. It can be seen how the 15 removed eigenvalues have actually very low values. We could have excluded even more eigenvalues without a great loss in data variance, but we decided to keep them in order not to lose any valuable information. Removing the less significant principal components helped reducing the computational complexity of further analysis.

Matrix **A** (see equation 1) was initialized with the first independent component and the deflation approach was chosen. The deflationary estimation of independent components consists in obtaining first one independent component (typically by maximizing a measure of non-Gaussianity) then estimating the second component, discarding the direction of the first one, and so on, repeating the procedure until all the independent components are obtained (Delfosse *et al.*, 1995). Typically this is done by constraining the search for new independent components to the space that is orthogonal to the already found components. We ordered the independent components and selected the first 10 independent components by means of the amount of data variance they retain and by the non-Normality of their distribution, as described by Liebermeister (2002). The robustness of our approach (caused by its SOM-based nature) makes it not sensitive to the exact number of independent components employed. Our choice was driven by the need not to include too many components that actually describe 'noise'.

Figure 4 shows the first five characteristic basis functions resulted from the ICA transform (rows of matrix **A**). It is interesting to observe that each of them seems to capture information that corresponds to a different subgroup of experiments. The three upper basis functions are more active during the cell-cycle experiments. The fourth shows an increased response during the sporulation and diauxic shift experiments, while the fifth is mainly active during the sporulation experiments.

Patterns having as elements the coefficients of the ten 'most important' independent components selected above were then used as input to our map. Therefore the input data matrix contained 6221 patterns of 10 elements (the representation of original gene expression profiles in the component space defined by the selected independent components). The algorithm selects for this dataset the model with a supervision parameter value $r_{su} = 0.6$. It

Copyright © 2005 John Wiley & Sons, Ltd. 

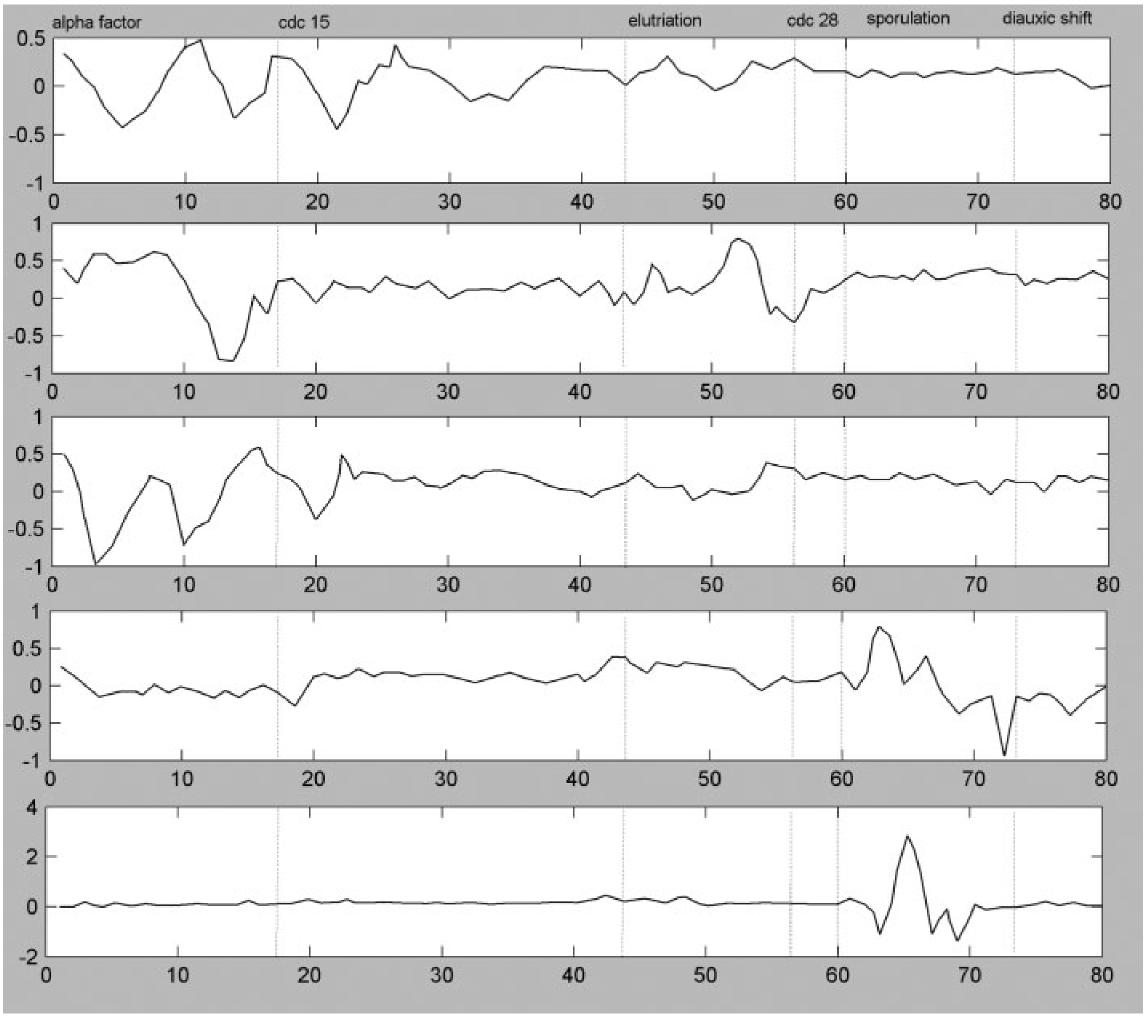

**Figure 4.** Five characteristic basis functions derived by independent component analysis. It can be observed how each of them is more active for a different subgroup of experiments. The three upper basis functions capture the information concerning mostly cell-cycle experiments (cell cycle $\alpha$-factor, cdc 15-based synchronization, elutriation and cdc-28), the fourth captures information regarding the sporulation and diauxic shift experiments, while the last one captures information mainly about the sporulation experiments. The cell cycle $\alpha$-factor (18 time points), the cdc 15-based synchronization (25 time points), the elutriation (14 time points) and the cdc 28 (3 time points) experiments were performed by Spellman *et al.* (1998), the sporulation experiment (13 time points) was performed by Chu *et al.* (1998), while the diauxic shift experiment was performed by DeRisi *et al.* (1997)

corresponds to a model with an acceptable classification performance ($ClassifPerf = 0.65$), small model complexity (map with reduced number of nodes) and a small rate of increase of the classification performance for further increased values of $r_{su}$ (see Table 2). Our approach does not set out to enforce a perfect classification but rather to perform exploratory analysis while making use of the existing supervised information. The relatively low rate of increase in the correct classification rate on the performance curve in Figure 5 indicates that further increasing the influence of the supervised information above the chosen $r_{su}$ value does not result in a significant increase of the classification performance, which may be due to overfitting. The SOM-based nature of our method enables data visualization during the learning process.

Copyright © 2005 John Wiley & Sons, Ltd. 

**Table 2.** The performance of the model for increasing $r_{su}$ parameter values and the corresponding number of map nodes

| $r_{su}$ | Number of nodes $K$ | Classification performance |
|---|---|---|
| 0 | 62 | 0.18 |
| 0.1 | 65 | 0.21 |
| 0.2 | 69 | 0.35 |
| 0.3 | 68 | 0.45 |
| 0.4 | 65 | 0.49 |
| 0.5 | 63 | 0.53 |
| 0.6 | 70 | 0.65 |
| 0.7 | 77 | 0.66 |
| 0.8 | 89 | 0.69 |
| 0.9 | 90 | 0.76 |
| 1 | 99 | 0.77 |

It should be noted that increasing the amount of supervised information used during learning (increasing $r_{su}$) does not necessary mean that the number of nodes should increase. In some cases supervised information helps in producing a more compact map.

### Exploratory data analysis experiment

In order to test how much the incorporation of *a priori* class information helps exploring unlabelled data, we unlabel 30% of the data in *dataset 1* and arbitrarily split the 30% in two equal subsets (subsets T and V, each containing 15% of *dataset 1*). We monitor the classification performance for subset V in two cases: when both T and V are unlabelled and separately, with the labels of T added back. In both cases, the remaining 70% of the data is labelled. The difference in the percentage of labels of V correctly induced shows how much the incorporation of *a priori* class knowledge (about T) helps in the exploratory analysis of V. The procedure is repeated for 50 random subsets and results of the experiment are presented in Table 3. As expected, with increasing values of the $r_{su}$ parameter, the effect of adding labels to the subset T is stronger. Overall, the

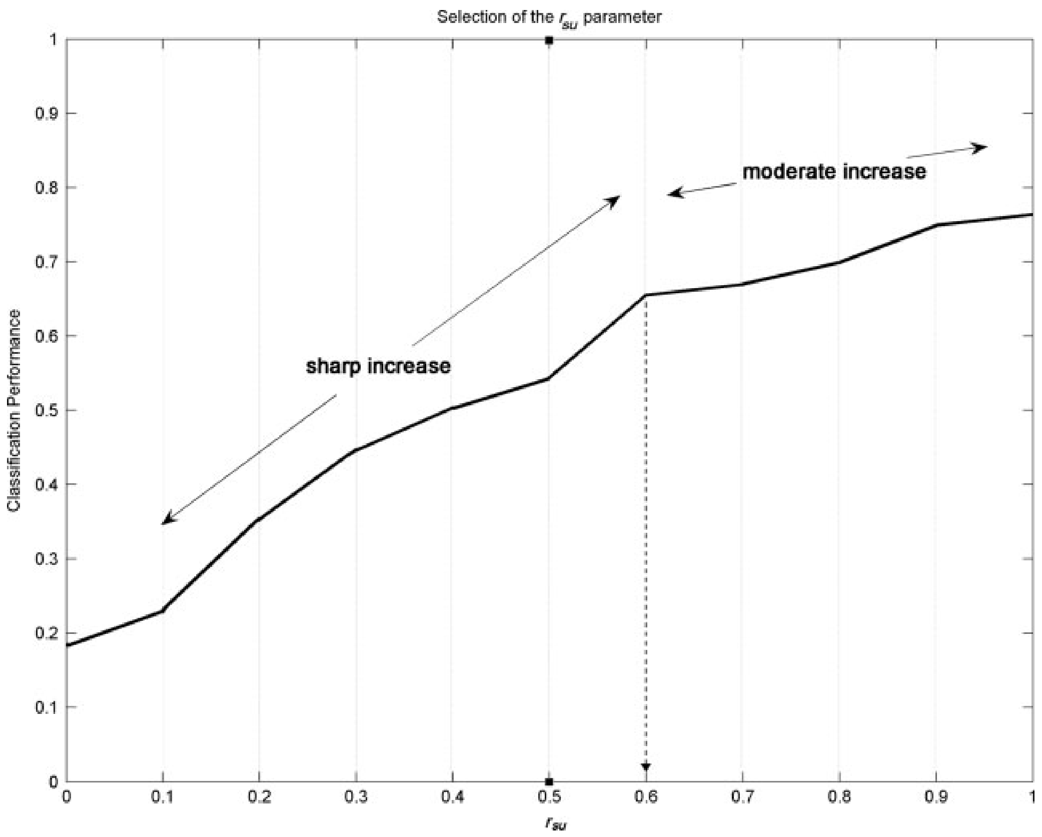

**Figure 5.** Selection of an appropriate $r_{su}$ parameter, corresponding to the model that makes most effective use of the supervised information. The classification performance should present a reduced rate of increase with values of $r_{su}$ higher than the chosen one

Copyright © 2005 John Wiley & Sons, Ltd. 

results prove the utility of our method as an exploratory analysis tool.

### Classification validity experiments

Another set of experiments we performed on *dataset 1* aimed at investigating the classification validity of our approach. Specifically, we selected six of the 19 top-level functional categories of the entire dataset, as marked in Table 1. A total of 2262 genes belong to these categories and the patterns corresponding to their representation in independent component space was used in subsequent analysis. Our selection objective was to include categories containing different number of genes (Metabolism contains 1059 genes, while just 59 genes represent the Signal Transduction Mechanisms category) such as to test our approach's performance when confronted to categories of a wide range of sizes. The decision to work on a subset of the data is motivated by the reduction of the large computational requirements and by obtaining a better control of the evaluation process.

We induce randomly a small number of errors in the original labelling and test to which extent the unsupervised drive of our algorithm can recover the original labelling. The incorrect labelled patterns should be assigned to a node of the correct class by the algorithm, if the unsupervised learning is predominant. Specifically, in the case of the categories presented above, we alter the labelling of around 10% of the patterns in each category and we monitor our algorithm's capacity of recovering the original classification by means of unsupervised learning. We repeat the procedure on random partitions of the set in order to obtain statistical confidence (the results in Table 4 are obtained for 50 repetitions). Indeed, the experiment shows that for small values of $r_{su}$ the original labelling is recovered, while increasing the contribution of the supervised information leads to patterns being assigned to the classes indicated by the wrong labelling.

Furthermore, Table 5 presents classification results of our approach (denoted as SOM-IC) compared to those of established methods, as well as our previous method (sNetSOM) from Mavroudi *et al.* (2002). As expected, the support vector machine (SVM) obtains the highest classification performance; however, our approach yields results competitive to other algorithms. All the algorithms were tested with a 10-fold cross-validation technique (Yeung *et al.*, 2001 a). Again as expected,

**Table 3.** Means $\pm$ standard errors of the exploratory data analysis experiment

| $r_{su}$ | Classification performance on subset V Subsets T and V unlabelled | Classification performance on subset V Only subset V unlabelled |
|---|---|---|
| 0 | $0.712 \pm 0.004$ | $0.707 \pm 0.003$ |
| 0.2 | $0.738 \pm 0.003$ | $0.758 \pm 0.002$ |
| 0.4 | $0.764 \pm 0.003$ | $0.802 \pm 0.003$ |
| 0.6 | $0.809 \pm 0.003$ | $0.883 \pm 0.002$ |
| 0.8 | $0.854 \pm 0.003$ | $0.914 \pm 0.003$ |
| 1 | $0.863 \pm 0.002$ | $0.943 \pm 0.002$ |

30% of the data in dataset 1 is unlabelled and split in two equal subsets, T and V, while the other 70% is left as in the original dataset. Subsequently, classification performance on the patterns from subset V is evaluated in two settings: with both subsets T and V unlabelled; or with only subset V unlabelled. The figures represent ratios of patterns belonging to subset V that are correctly classified, averaged over 50 random experiment reruns and their respective standard errors (we compute the standard error as the standard deviation in the sample divided by the square root of the sample's size).

**Table 4.** Means $\pm$ standard errors of class recovery ratios from experiments performed on data with altered labelling

| $r_{su}$ | Metabolism (1059) | Cell fate (423) | Cellular communication (59) | Cell cycle (620) | Control of cellular organization (205) | Transport facilitation (305) |
|---|---|---|---|---|---|---|
| 0 | $0.813 \pm 0.003$ | $0.906 \pm 0.003$ | $0.823 \pm 0.003$ | $0.801 \pm 0.003$ | $0.861 \pm 0.003$ | $0.883 \pm 0.003$ |
| 0.2 | $0.762 \pm 0.003$ | $0.818 \pm 0.003$ | $0.738 \pm 0.003$ | $0.758 \pm 0.003$ | $0.813 \pm 0.003$ | $0.786 \pm 0.003$ |
| 0.4 | $0.676 \pm 0.003$ | $0.733 \pm 0.003$ | $0.614 \pm 0.003$ | $0.623 \pm 0.003$ | $0.729 \pm 0.003$ | $0.635 \pm 0.003$ |
| 0.6 | $0.531 \pm 0.003$ | $0.658 \pm 0.002$ | $0.523 \pm 0.003$ | $0.554 \pm 0.003$ | $0.564 \pm 0.003$ | $0.542 \pm 0.003$ |
| 0.8 | $0.484 \pm 0.003$ | $0.513 \pm 0.002$ | $0.388 \pm 0.003$ | $0.456 \pm 0.003$ | $0.442 \pm 0.002$ | $0.441 \pm 0.002$ |
| 1.0 | $0.292 \pm 0.002$ | $0.417 \pm 0.002$ | $0.311 \pm 0.003$ | $0.383 \pm 0.003$ | $0.384 \pm 0.003$ | $0.388 \pm 0.002$ |

Class recovery ratios represent the percentage of successful label corrections performed on the mislabelled patterns. The results are averaged over 50 random repetitions and next to them are presented the respective standard errors (we compute the standard error as the standard deviation in the sample divided by the square root of the sample's size). It can be noticed that for small values of $r_{su}$ the algorithm recovers to a higher degree the original labelling, while high values of $r_{su}$ force the use of altered labels.

Copyright © 2005 John Wiley & Sons, Ltd.

**Table 5.** Comparative classification performance of the current approach (denoted by SOM-IC) and with results for low, optimal and high degree of influence of *a priori* supervised information, along with a previous approach of Mavroudi *et al.* (2002)*

| Functional class | Method | Precision | Recall |
|---|---|---|---|
| Cell fate | SOM-IC ($r_{su} = 0.1$) | 0.57 ÷ 0.01 | 0.64 ÷ 0.01 |
| | SOM-IC_no_ICA($r_{su} = 0.1$) | 0.51 ÷ 0.02 | 0.60 ÷ 0.02 |
| | SOM-IC ($r_{su} = 0.6$) | 0.68 ÷ 0.01 | 0.71 ÷ 0.01 |
| | SOM-IC_no_ICA($r_{su} = 0.6$) | 0.67 ÷ 0.01 | 0.71 ÷ 0.01 |
| | SOM-IC ($r_{su} = 1$) | 0.73 ÷ 0.01 | 0.75 ÷ 0.01 |
| | SOM-IC_no_ICA($r_{su} = 1$) | 0.73 ÷ 0.01 | 0.73 ÷ 0.01 |
| | sNetSOM ($r_{su} = 1$) | 0.44 ÷ 0.01 | 0.57 ÷ 0.02 |
| | sNetSOM ($r_{su} = 10$) | 0.65 ÷ 0.01 | 0.71 ÷ 0.02 |
| | sNetSOM ($r_{su} = 20$) | 0.70 ÷ 0.01 | 0.75 ÷ 0.02 |
| | Naïve Bayes | 0.41 ÷ 0.01 | 0.43 ÷ 0.01 |
| | SVM | 0.96 ÷ 0.01 | 0.83 ÷ 0.01 |
| Cell cycle and DNA processing | SOM-IC ($r_{su} = 0.1$) | 0.49 ÷ 0.01 | 0.75 ÷ 0.01 |
| | SOM-IC_no_ICA($r_{su} = 0.1$) | 0.48 ÷ 0.02 | 0.71 ÷ 0.02 |
| | SOM-IC ($r_{su} = 0.6$) | 0.59 ÷ 0.01 | 0.77 ÷ 0.01 |
| | SOM-IC_no_ICA($r_{su} = 0.6$) | 0.58 ÷ 0.01 | 0.77 ÷ 0.01 |
| | SOM-IC ($r_{su} = 1$) | 0.66 ÷ 0.01 | 0.82 ÷ 0.01 |
| | SOM-IC_no_ICA($r_{su} = 1$) | 0.66 ÷ 0.01 | 0.81 ÷ 0.01 |
| | sNetSOM ($r_{su} = 1$) | 0.47 ÷ 0.02 | 0.66 ÷ 0.01 |
| | sNetSOM ($r_{su} = 10$) | 0.56 ÷ 0.01 | 0.75 ÷ 0.01 |
| | sNetSOM ($r_{su} = 20$) | 0.65 ÷ 0.01 | 0.80 ÷ 0.02 |
| | Naïve Bayes | 0.37 ÷ 0.01 | 0.67 ÷ 0.01 |
| | SVM | 0.97 ÷ 0.01 | 0.83 ÷ 0.01 |
| Control of cellular organization | SOM-IC ($r_{su} = 0.1$) | 0.62 ÷ 0.01 | 0.53 ÷ 0.01 |
| | SOM-IC_no_ICA($r_{su} = 0.1$) | 0.54 ÷ 0.01 | 0.51 ÷ 0.02 |
| | SOM-IC ($r_{su} = 0.6$) | 0.71 ÷ 0.01 | 0.57 ÷ 0.01 |
| | SOM-IC_no_ICA($r_{su} = 0.6$) | 0.67 ÷ 0.01 | 0.56 ÷ 0.01 |
| | SOM-IC ($r_{su} = 1$) | 0.79 ÷ 0.01 | 0.63 ÷ 0.01 |
| | SOM-IC_no_ICA($r_{su} = 1$) | 0.76 ÷ 0.01 | 0.62 ÷ 0.01 |
| | sNetSOM ($r_{su} = 1$) | 0.49 ÷ 0.02 | 0.48 ÷ 0.02 |
| | sNetSOM ($r_{su} = 10$) | 0.64 ÷ 0.02 | 0.55 ÷ 0.02 |
| | sNetSOM ($r_{su} = 20$) | 0.70 ÷ 0.02 | 0.61 ÷ 0.01 |
| | Naïve Bayes | 0.35 ÷ 0.01 | 0.66 ÷ 0.01 |
| | SVM | 0.86 ÷ 0.01 | 0.72 ÷ 0.01 |

* Denoted by sNet-SOM, which has optimal $r_{su} = 10$, as well as established classification methods. SOM-IC_no_ICA denotes the experiment performed with the current approach directly on gene expression profiles (before applying ICA). The last two columns present the averages of precision and recall values and their observed variance over the 10-fold cross-validation. The median absolute deviation (MAD) quantifies the variance of the results from cross-validation and is calculated as the median of the absolute-value distances of the points about the median, multiplied by the constant 1.4286, i.e. adjusted for asymptotic normal convergence, e.g. MAD = 1.4286· median $|x - \text{median}(x)|$. Precision = TP/(TP + FP), while Recall = TP/(TP + FN). TP, true positives; TN, true negatives; FP, false positives; FN, false negatives.

classification performance is influenced by values taken by the $r_{su}$ parameter. Using the available supervised information (by increasing the value of $r_{su}$) results in improved classification rates. The classification results of our method using as input the gene expression profiles (before applying ICA; entries SOM-IC_no_ICA in Table 5) prove that employing profiles' representations in the independent component space improves the performance. However, for high values of $r_{su}$, the improvement becomes insignificant. This may be due to the fact that the supervised information used determines the algorithm to take decisions primarily based on the class information and not on the pattern similarities.

As a confirmation of other recent studies (Saidi *et al.*, 2004) which show the benefits of unsupervised clustering in the component space, we present in the Supplementary Material results of

Copyright © 2005 John Wiley & Sons, Ltd. 

the experiments testing our approach's handling of pure unsupervised problems, both using the original gene profiles and their representations in the component space.

### Dataset 2

The dataset consists of expression profiles of 2476 yeast genes samples at different time points and during eight experimental settings (Brown *et al.*, 1999), in total 79 measurements for each gene. Six functional classes are contained in the dataset and they were obtained again from the MIPS Yeast Genome Database. The first five classes [tricarboxylic acid cycle (TCA), respiration chain complexes, cytoplasmic ribosomes, proteasomes and histones] represent biological categories of genes and are therefore expected to present a similar expression variation. The sixth (helix–turn–helix proteins) is not a functional class but genes included in this group have in common that they code for the helix–turn–helix structural motif; in this analysis they are used as a control set.

We evaluated the dispersion of class representation over the nodes of the final map configuration by measuring the entropy of class representation. We expect this measure to be high in the case of helix–turn–helix class, due to the heterogeneity of the respective gene patterns. The relatively high entropy values for the TCA and Respiration classes may be explained by their less homogeneous structure (the fact that these classes accumulate patterns belonging to other functional classes is caused by the low degree of similarity among their patterns). As in the previous experiment, we have run the algorithm both on the original gene expression data and on the ICA-derived patterns. The results obtained are presented in Table 6. The higher entropy values obtained for the map structure corresponding to the analysis performed directly on gene expression profiles suggest a higher dispersion of class representation and therefore less homogeneous clusters. However, the high degree of supervised information employed may be, as in the experiment above, the cause for the relatively low difference in entropies for the algorithm running on the two types of data. The map structure studied is the one corresponding to the model with an optimal value of $r_{su} = 0.6$.

**Table 6.** Values of the entropy of class representation for the classes present in dataset 2

| Class | Entropy (component space data) | Entropy (original gene experiment) |
|---|---|---|
| Tricarboxylic acid pathway (TCA) | 1.96 | 1.99 |
| Respiration chain complexes | 1.82 | 1.89 |
| Cytoplasmic ribosomal proteins | 1.21 | 1.28 |
| Proteasome | 0.51 | 0.53 |
| Histones | 0.60 | 0.64 |
| Helix–turn–helix | 2.78 | 2.91 |

The first column contains the functional classes, the second column contains results from analysis performed with patterns from the independent component space, while results corresponding to analysis performed directly on gene expression data are presented in the third column. As expected, the entropy measure for the helix–turn–helix is high, reflecting the heterogeneity of the patterns representing this class.

### Conclusions

We have introduced a self-growing adaptive network, which aims to overcome the drawbacks of current clustering algorithms for gene expression data by swiftly integrating unsupervised and supervised learning. The algorithm adaptively determines the number of clusters with a dynamic expansion process based on the principle of local error accumulation. It starts with a small number of nodes and grows to represent the input data. The expansion algorithm prefers to grow new nodes in the neighbourhood of boundary nodes, with a mechanism for whole-column insertion being provided in order to deal with the cases when large maps need to be expanded from a node deep within its interior. The level of expansion is determined automatically, based on a statistical confidence level of non-randomness chosen by the data analyst.

The method builds on our previous work (Mavroudi *et al.*, 2002) by efficiently incorporating independent component analysis in a drive to find a more meaningful representation of the data. The method yields an interesting outcome, highlighting particular biological processes as well as representing the data in a biologically sensible way. Also, compared to our previous work, the convergence criteria are improved and more thoroughly defined. Comparative evaluation of the current method as a reclassification device proves it to be superior

Copyright © 2005 John Wiley & Sons, Ltd. 

to our previous approach, with both the incorporation of ICA into our analysis and the enhancements brought to the SOM-based algorithm contributing to this improvement.

Our approach efficiently uses supervised information, whenever available, and is able to deal with incomplete or unreliable functional labelling. Additionally, the method allows enhanced representation of rare classes and successfully handles multi-labelled gene expression profiles.

The resulting clusters are enriched with functionally related genes. Of course, since we use functional information in our supervised learning, this is not surprising. We have to stress though, that the purpose of our algorithm is not to classify the gene patterns according to existing classification, but to include the existing knowledge, which is known to be evolving and incomplete, in order to cluster (reclassify) the genes and to assign a function to unlabelled genes. At the same time, our method proves to be a reliable tool in exploratory analysis, the presented results underlining the positive effect of incorporating *a priori* class knowledge when exploring unlabelled data.

In future work we intend to further analyse our results from a biological perspective. Especially, we intend to investigate whether, on distinct subsets of clusters that all correspond to a particular top-level functional category, we possibly get a mapping of genes that belong to distinct functional subcategories. Furthermore, it is known that genes are co-expressed with different groups of genes, each group being controlled by a distinct regulatory mechanism, in response to different environmental conditions of the cell (Gash *et al.*, 2002). We intend to uncover the characteristic 'features' of each cluster (i.e. the experiments on which the grouping of genes is primarily based), in order to elucidate the relationships between gene classes and experimental conditions. In this way we expect to obtain biological knowledge about multi-labelled genes, e.g. to uncover the reasons that cause the same gene to be involved in several processes.

### Acknowledgements

The authors would like to thank the European Social Fund (ESF), the Operational Program for Educational and Vocational Training II (EPEAEK II), and particularly the Program IRAKLEITOS, for financially supporting this work.

### References

Alahakoon D, Halgamuge SK, Srinivasan B. 2000. Dynamic self-organizing maps with controlled growth for knowledge discovery. *IEEE Trans Neural Netw* **11**: 601–614.

Azuaje F. 2001. A computational neural approach to support the discovery of gene function and classes of cancer. *IEEE Trans Biomed Eng* **48**: 332–339.

Brazma A, Vilo J. 2000. Gene expression data analysis. *FEBS Lett* **480**: 17–24.

Bittner M, Meltzer P, Chen Y, *et al.* 2000. Molecular classification of cutaneous malignant melanoma by gene expression profiling. *Nature* **406**: 536–540.

Brown MPS, Grundy WN, Lin D, *et al.* 1997. Knowledge-based analysis of microarray gene expression data by using support vector machines. *Proc Natl Acad Sci USA* **97**: 262–267.

Campos M, Carpenter GA. 2001. S-TREE: self-organizing trees for data clustering and online vector quantization. *Neural Netw* **14**: 505–525.

Cheng G, Zell A. 2001. Externally growing cell structures for data evaluation of chemical gas sensors. *Neural Comput Appl* **10**: 89–97.

Cheung VG, Morley M, Aguilar F, *et al.* 1999. Making and reading microarrays. *Nature Genet* **21**(suppl).

Chu S, DeRisi JL, Eisen M, *et al.* 1998. The transcriptional program of sporulation in budding yeast. *Science* **282**: 699–705.

Delfosse N, Loubaton P. 1995. Adaptive blind separation of independent sources: a deflation approach. *Signal Proc* **45**: 59–83.

DeRisi JL, Iyer VR, Brown PO. 1997. Exploring the metabolic and genetic control of gene expression on a genomic scale. *Science* **278**: 680–686.

Eisen MB, Spellman PT, Patrick OB, Botstein D. 1998. Cluster analysis and display of genome-wide expression patterns. *Proc Natl Acad Sci USA* **95**: 14 863–4868.

Friedman N, Linial M, Nachman I, D'Peier. 2000. Using Bayesian networks to analyse expression data: *J Comp Biol* **7**: 601–620.

Fritzke B. 1995. Growing Grid — a self-organizing network with constant neighborhood range and adaptation strength. *Neural Proc Lett* **2**: 9–13.

Gash PA, Eisen BM. 2002. Exploring the conditional co-regulation of yeast gene expression through fuzzy K-means clustering. *Genome Biol* **3**: **http://genomebiology.com/2002/3/II/research/0059**

Hastie T, Tibshirani R, Botstein D, Brown P. 2001. Supervised harvesting of expression trees. *Genome Biol* **2**: **http://genomebiology.com/2001/2/I**

Haykin S. 1999. *Neural Networks*, 2nd edn. Prentice Hall International: Upper Saddle River, NJ.

Herrero J, Valencia A, Dopazo J. 2001. A hierarchical unsupervised growing neural network for clustering gene expression patterns. *Bioinformatics* **17**: 126–136.

Hyvarinen A, Oja E. 1997. A fast fixed point algorithm for independent component analysis. *Neural Comput* **9**: 1483–1492.

Hyvarinen A. 1999. Fast and robust fixed-point algorithms for independent component analysis. *IEEE Trans Neural Netw* **10**: 626–634.

Karhunen J, Hyvarinen A, Vigario R, *et al.* 1997. Applications of neural blind source separation to signal and image processing. Proceedings of the IEEE 1997 International Conference on

Acoustics, Speech and Signal Processing (ICASSP'97), Munich, Germany.

Kohane IS, Kho AT, Butte AJ. 2003. *Microarrays for an Integrative Genomics: A Bradford Book.* MIT Press, Cambridge, MA.

Kohonen T. 2001. *Self-organized Maps*, 3rd edn. Springer-Verlag, Secaucus, NJ.

Kreil DP, MacKay DJC. 2003. Reproducibility assessment of independent component analysis of expression ratios from DNA microarrays. *Comp Funct Genom* **4**: 300–317.

Lee S-I, Batzoglou S. 2003. Applications of independent component analysis to microarrays. *Genome Biol* **4**: R76.1.

Liebermeister W. 2002. Linear modes of gene expression determined by independent component analysis. *Bioinformatics* **18**: 51–60.

Mavroudi S, Papadimitriou S, Bezerianos A. 2002. Gene expression analysis with a dynamically extended self-organized map that exploits class information. *Bioinformatics* **18**: 1446–1453.

Moreau Y, De Smet F, Thijs G, *et al*. 2002. Functional bioinformatics of microarray data: from expression to regulation. *Proc IEEE* **90**: 1722–1743.

Morgan BJT, Ray APG. 1995. Non-uniqueness and inversions in cluster analysis. *Appl Statist* **44**: 117–134.

Papadimitriou S, Mavroudi S, Vladutu L, Bezerianos A. 2001. Ischemia detection with a self-organizing map supplemented by supervised learning. *IEEE Trans Neural Netw* **12**: 503–515.

Rauber A. 1999. LabelSOM: on the labelling of self-organizing maps. *Proceedings of the International Joint Conference on Neural Networks (IJCNN '99)* **5**: 3527–3532.

Sable CL, Hatzivassiloglou V. 1999. Text-based approaches for the categorization of images. *Proceedings of the 3rd Conference on Research and Advanced Technology for Digital Libraries, Paris*: 19–38.

Saidi SA, Holland CM, Kreil DP, *et al*. 2004. Independent component analysis of microarray data in the study of endometrial cancer. *Oncogene* **23**: 6677–6683.

Si J, Lin S, Vuong MA. 2000. Dynamic topology representing networks. *Neural Netw* **13**: 617–627.

Spellman PT, Sherlock G, Iyer VR, *et al*. 1998. Comprehensive identification of cell cycle-regulated genes of the yeast *Saccharomyces cerevisiae* by microarray hybridization. *Mol Biol Cell* **9**: 3273–3297.

Tamayo P, Slonim D, Mesirov J, *et al*. 1999. Interpreting patterns of gene expression with self-organizing maps: methods and application to hematopoietic differentiation. *Proc Natl Acad Sci USA* **92**: 2907–2912.

Troyanskaya O, Cantor M, Shelock G, *et al*. 2001. Missing value estimation methods for DNA microarrays. *Bioinformatics* **17**: 520–525.

Yeung KY, Haynor DR, Ruzzo WL. 2001a. Validating clustering for gene expression data. *Bioinformatics* **17**: 309–318.

Yeung KY, Ruzzo WL. 2001b. Principal component analysis for clustering gene expression data. *Bioinformatics* **17**: 763–774.

Van Hulle NM. 2002. Joint entropy maximization in Kernel-based topographic maps. *Neural Comput* **14**: 1887–1906.

Vesanto JA. 2000. Clustering of the self-organized map. *IEEE Trans Neural Netw* **11**: 586–600.

Copyright © 2005 John Wiley & Sons, Ltd. 
